# Results of a hydroxyapatite-coated femoral stem (Corail) in Chinese: a minimum 10-year follow-up

**DOI:** 10.1186/s40064-016-3656-5

**Published:** 2016-11-15

**Authors:** Jianda Xu, Zikang Xie, Jianning Zhao, Yi Gao, Hong Zhao, Libo Peng, Yuxing Qu

**Affiliations:** 1 Department of Orthopaedics, Changzhou Traditional Chinese Medical Hospital, Affiliated to Nanjing University of Traditional Chinese Medicine, 25 North Heping Road, Changzhou, 213000 Jiangsu China; 2 Nanjing Jinling Hospital, 305 East Zhongshan Road, Nanjing, 210002 Jiangsu China

## Abstract

**Background:**

Due to the adverse effects of cemented hip arthroplasty, uncemented stems with hydroxyapatite (HA) coating reduces these risks and enhanced integration. The concept of an extensive HA coating for the fixation of a tapered femoral stem (Corail^®^) was introduced, which can achieve durable biological fixation and preserve normal periprosthetic bone activity. Here we describe the clinical and radiological outcome in patients with the Corail^®^ stem.

**Methods:**

92 total hip replacements in 81 patients using the Corail^®^ stem were followed-up. 47 patients were women, and the mean age at surgery was 62.9 ± 8.7 (34–71) years. The indications included: osteoarthritis of the hip (71.1%), avascular necrosis (13.6%), femur neck fractures in elderly (9.7%) and post-traumatic osteoarthritis (6.8%).

**Findings:**

Eight patients died during follow-up. The revision was only found in two patients due to line wear and resulted in an 10-year Kaplan–Meier estimated overall survival rate of 97.83%. The clinical results were good, with a mean Harris hip score of 92.3 ± 5.6 (72–100). The mean total Merle d’Aubigné and Postel score was 6.8 ± 0.5 pre-operatively and 16.1 ± 1.4 at latest follow-up. All unrevised implants were radiographically stable, with a mean liner wear of 0.07 mm/year.

**Conclusion:**

This long-term analysis confirmed the durability of the functional and radiographic results. Our findings suggest the long-term results of Corail^®^ HA-coated stem are more satisfactory which is preferable to any other system.

## Background

Because of the tremendous evolutions in the last two decades, cementless fixation of the femoral component has becoming an gold standard in total hip arthroplasty (THA). Various stems available are coated by porous coating, grit blasting or hydroxyapatite, which supply initial press-fit fixation and later biologic fixation (Reigstad et al. 2008; Shetty et al. 2005; Aldinger et al. 2003).

The concept of an extensive HA coating for the fixation of a tapered femoral stem (Corail^®^) was introduced, which can achieve durable biological fixation and preserve normal periprosthetic bone activity (Chambers et al. 2007). It has gained popularity among orthropaedics surgeons.

Hydroxyapatite (HA) as a coating is one of the most generally accepted and commercialised bioactive materials. The Corail^®^ stem is made of forged titanium alloy (TiAl_6_V_4_). The combination of a tapered titanium stem with an extensive coating may increase fixation with harmonious stress distribution. The proximal part is flared in the sagittal and the coronal plane to provide three-dimensional stabilisation in the metaphyseal area. The distal portion has a tapered design, to produce a stiffness gradient and to avoid medullary canal blocking. The HA coating is applied to the entire stem in order to prevent the release of metal ions, to provide for maximum osseointegration at the interface and to prevent the interposition of a fibrous membrane around the distal portion of the stem (Vidalain and ARTRO Group 1999).

The short-term results are good with respect to relief from pain and function, but few long-term results with a follow-up of more than 10 years are reported. The aim of this study was prospectively to evaluate the long term clinical and radiological outcome of the extensive HA coating Corail^®^ stem.

## Patients and methods

The study protocol was approved by the Ethics Review Committee of Changzhou Traditional Chinese Medical Hospital, affiliated to Nanjing University of Traditional Chinese Medicine. Consents were obtained before all study. The study was carried out in accordance with the approved guidelines.

Between Oct. 2001 and Oct. 2003, we performed 92 THA in 81 patients using the extensive HA coating Corail^®^ stem (Depuy, USA). The collected data were successively registered in paper. There were 34 males (42.0%) and 47 females (58.0%) with an average age of 62.9 ± 8.7 years (range 34–71 years).

All operations were undertaken by one experienced orthropaedic surgeon with a specialist in arthroplasty. Template was used preoperatively to determine the cup size, the femoral size and the neck geometry. Surgery was performed under epidural anesthesia or general anaesthesia, and the bilateral procedures were performed in sequence under the same anaesthesia. Trial was done with the broach and appropriate trial head until a stable construct was achieved. The acetabular component (28 mm head) was implanted with a neck length varying from 1.5 to 8.5 mm. The process of rehabilitation was adjusted to each patient’s clinical course, early and full weight-bearing was encouraged after surgery.

Follow-up duration was defined as the date of operation to the date of last follow-up. The minimum follow-up was 10 years (mean 11.3 ± 1.5 years, 10–13 years). The survival curves were drawn according to the Kaplan–Meier method, and the survivorship was defined as revision for any reasons.

The clinical and radiological review was taken at 6 weeks, 3, 6, 12 months and then annually. At each review, the following parameters were used: the visual analogue scale for pain (VAS, 0 representing “no pain” and 10 representing “the worst pain ever” (Stubbs 1979), the Merle d’Aubigné and Postel (MDP) score (Merle d’Aubigne and Postel 1954) and function (Harris Score) (Harris 1969) and complications. The radiological were obtained from an standard anteroposterior view of the pelvis and a lateral view of the hip. The radiographs were analysed by two authors who were blinded to the clinical result. We noted the radiolucencies around the stem in each of the seven Gruen zones (Gruen et al. 1979), the presence or absence of an osteoblastic reaction at the tip suggesting bone ingrowth on to the HA coated stem and subsidence of the stem (Stem fixation). Subsidence was defined as comparison between the radiographs taken immediate post operatively and at last fllow-up. The implant subsidence was measured the change in the vertical distance between the superior tip of the greater trochanter and the lateral shoulder of the prosthesis (Engh et al. 1990).

## Results

There was no case of infection. Dislocation occurred in 2hips, which were reduced under anaesthesia and immobilised in skin traction for 6 weeks without further recurrence. Deep-vein thrombosis was diagnosed in 2 patients. One periprosthetic fracture following a fall occurred at 3 years and got operation with a locked plate. No other complications of surgery were recorded.

**Fig. 1:**
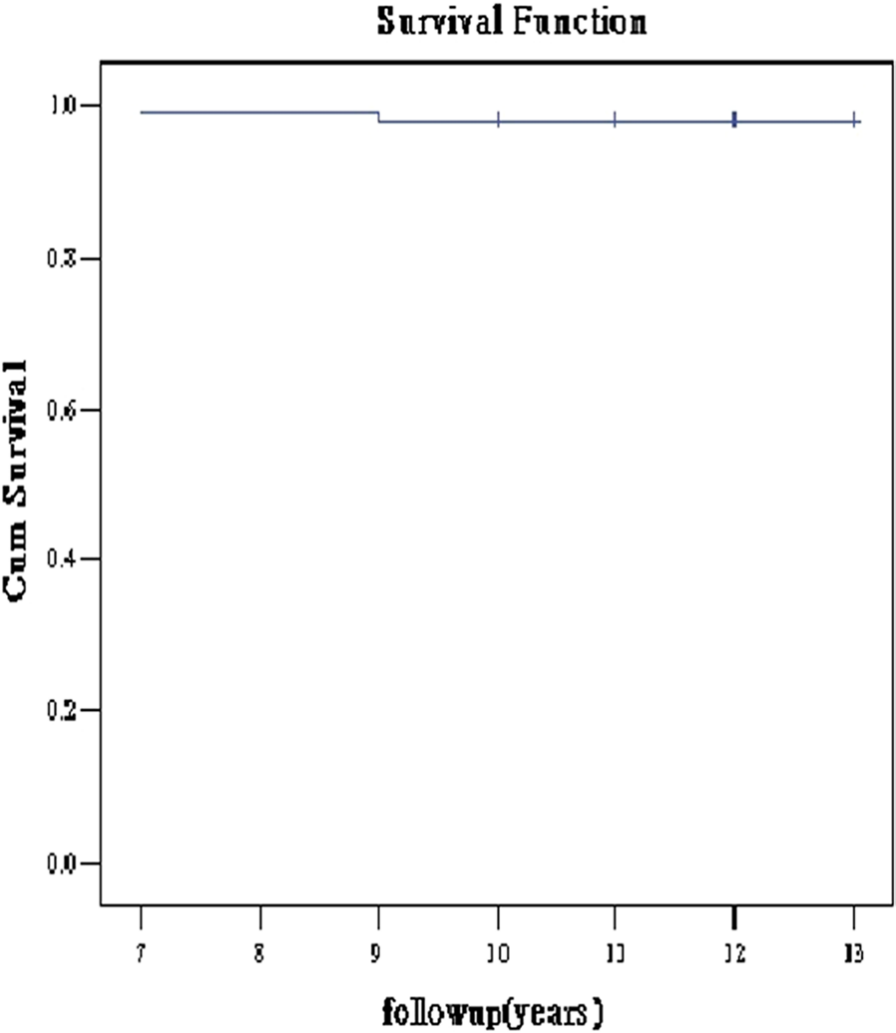
Kaplan–Meier estimated overall survival curves, showing the rate of survival of the 92 THAs

The revision was only found in 2 hips (34 and 47 years old) due to line wear and resulted in an 10-year Kaplan–Meier estimated overall survival rate of 97.83% (Fig. 1). The survival rate of the femoral stem was 100%. All unrevised implants were radiographically stable, with a mean liner wear of 0.07 mm/year.

### Clinical results

The clinical results were good, with a mean Harris hip score of 92.3 ± 5.6 (72–100). There were 73 patients (81 hips) were still alive and all were seen at latest follow-up. The Merle d’Aubigné and Postel score improved satisfactorily in all patients, and the mean total score was 6.8 ± 0.5 pre-operatively and 16.1 ± 1.4 at last follow-up. Each part of the score showed improvement (Table 1).

**Table 1:** The Merle d’Aubigné and Postel score

	Mean pre-operative	Mean post-operative
Pain	2.1 ± 0.6	5.7 ± 0.2
Mobility	2.5 ± 0.7	5.1 ± 0.6
Ability to walk	2.3 ± 0.3	5.5 ± 0.4
Total	6.8 ± 0.5	16.1 ± 1.4

No patient complained of anterior thigh pain. The mean VAS score was 1.2 ± 0.612, and 57 (70.4%) hips had no pain, 22 (27.2%) only slight or occasional pain. Only 2 (2.4%) had moderate pain on walking or at all times. One patient complaining constant pain in the operative leg with a visual analogue pain score of 6 had lumbar intervertebral disc herniation and healed after operation.

### Radiological results

In all postoperative radiographic films, the stems were usually centered or in slight varus and the limb length discrepancies were corrected. Radiolucencies around the acetabulum were found in 5 hips, with 4 in one zone (80%) and 1 in two zones (20%). No correlation was found between the radiolucencies and clinical outcome measure.

**Fig. 2:**
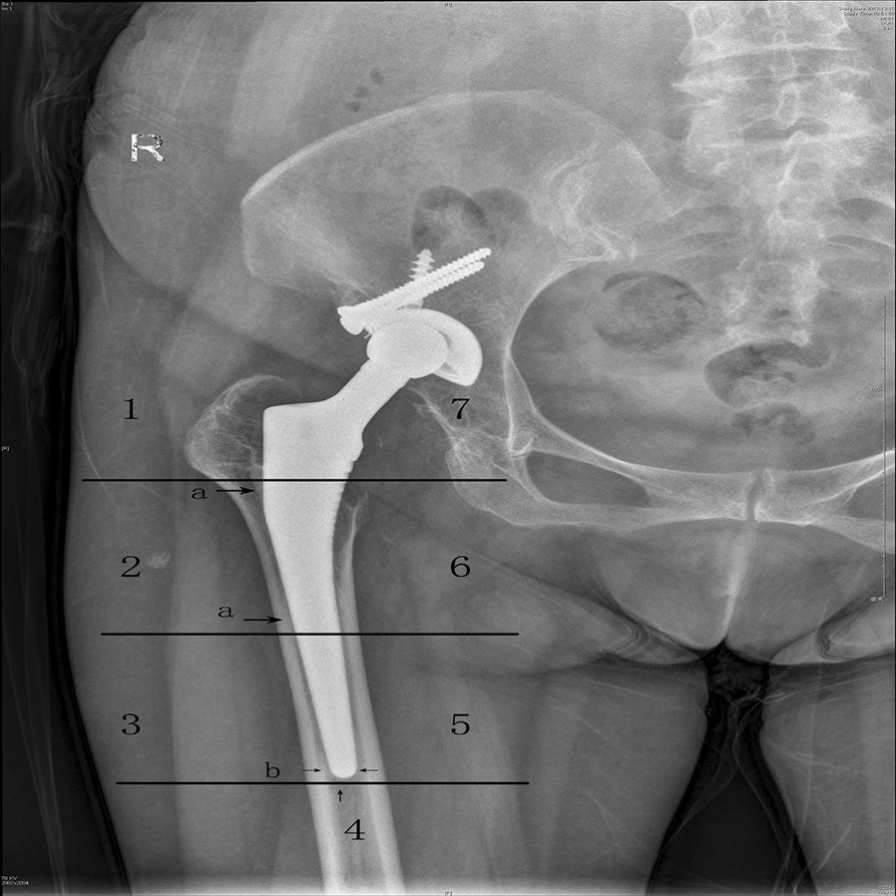
With the use of the zones described by Cruen et al., radiographs showing (*a*) spot weld and (*b*) pedestal formation around the femoral stem

There was no radiolucent line around the stem in any zone. Osteoblastic reaction was observed around stem in all stems, particularly in the 2–6 zones (Fig. 2). All the stems were excellent stable with no evidence of pedestal formation or radiolucent line. That suggests that distal fixation does not lead to stress shielding proximally.

## Discussion

Our aim was to evaluate accurate details by following-up of Chinese patients using Corail^®^ stem, a full extensive HA-coated femoral stem. Our series showed a survival rate of 100% of Corail^®^ stem at 10 years and an overall survival rate of 97.83%, equal to other published survival of HA-coated femoral components (Shetty et al. 2005; Vidalain 2011). J.A. Epinette reported that excellent results from the partially coated HA Ominifit stem with a survival rate of 99.20% at 17-year follow-up. Compared with porous alone femoral stems, HA stems demonstrated better result with less adverse effects (Epinette and Manley 2008). Sariali et al. (2012) reported that the survivalship of anatomic cementless stem (SPS) was 100% considering stem revision for aseptic loosening as the end point. Antonio Herrera et al. reported their surgical experience with more than 4000 cementless hydroxyapatite coated hip prostheses (the ABG I and ABG II prosthesis an anatomical HA-coated stem with press-fit metaphyseal fixation, Stryker) since 1990. The survival at 10-year follow-up was 97.1% in ABG I, while a mean of 11.3 years follow-up was 98.30% in ABG II (Herrera et al. 2015). All these results confirmed the long-term stability of HA-coated prostheses.

The Merle d’Aubigné and Postel score improved satisfactorily in all patients, and the mean total score was 6.8 ± 0.5 pre-operatively and 16.1 ± 1.4 at latest follow-up. Each part of the score showed improvement. The mean Harris hip score was 92.3 ± 5.6 (72–100) at last follow-up. To our opinion, the proximal flared design of Corail^®^ stem increases three dimensional metaphyseal femoral fit and avoid stress-shielding. Macrotextural features (horizontal and vertical grooves) enhance primary mechanical stability. The stability was achieved primarily through mechanical fixation (tight press-fit into the bone) and then secondarily through biological fixation (biological anchoring in bone), which is needed for a long-term survival of the prosthesis (Engh et al. 1987). However, the ARTRO Group affirmed that secondary fixation cannot take place unless primary mechanical fixation has been achieved (Vidalain and ARTRO Group 1999). It demonstrates early stability was essential for better rehabilitation (Kärrholm et al. 1994). Early peri-operative rehabilitation is pivotal for accelerated recovery and reduced the hospital length of stay after THA (Minns Lowe et al. 2009). The patients can early return to normal activities with absence of pain.

There was no patient in our series complaining of anterior thigh pain, in contrast with reports of other cementless femoral components without HA-coating (McNally et al. 2000; Engh and Massin 1989). The cause of this thigh pain had been considered to be multifactorial. M. Rokkum et al. reported an excellent clinical result of 100 consecutive entirely hydroxyapatite-coated hip arthroplasties in 86 patients. All patients had no thigh pain. They believed that all components were bonded directly to bone, promoted by the reliable primary fixation and the osteoconductive effect of HA (Rokkum and Reigstad 1999). The extensive HA coating and proximal flared design has the same function. And the diaphyseal part of the Corail stem offers a press fit property, as comfortable transition between the metaphyseal and diaphyseal part. Osteoblastic reaction was observed around stem, particularly in the 2–6 zones in our series. The distal stem fix indicated a shift from proximal to distal loading and ensured fewer incidences of thigh pain, similar with the previous results (Faraj and Yousuf 2005).

HA brings a chemical fixation between bone and implants, which can be resorped and replaced by bone within 16 weeks after implantation (Søballe et al. 1993). This phenomenon is a continuous, reproducible and reliable integration process. That means HA generates ‘spot welding’ of bone to the prosthesis, and impedes the access of polyethylene debris to the interface to prevent imminent or potential failure (Donnelly et al. 1997). As resorption of the HA coating, the new formed bone will replaces with a higher percentage. Complete osseointegration isn’t requisite, biological anchoring in bone can supply a sufficient stable fixation (Tonino et al. 2009). Young and very active patients could be surgical indications also (Wangen et al. 2008). Loupasis et al. (1998), in a 6 year follow-up study of 45 patients under the age of 50, reported no revision for aseptic loosening or evidence of stem loosening. What’s more, all the stems were excellently stable with no evidence of pedestal formation or radiolucent line. Ulivi et al. (2013) demonstrated that HA-coated implants have a higher long-term survival even in elderly patients. That suggests that distal fixation does not lead to stress shielding proximally.

In present group, the revision was only found in two patients (34 and 47 years old) due to line wear, which implied the age at the time of surgery maybe a significant influence factor for prosthesis revision. The previous reports have shown that higher rate of THR failure was associated with a higher proportion of PE wear (Reikeras and Gunderson 2002). Ohnsorge et al. (2006) considered the reason of higher proportion of failure was not solely poor PE quality, but also poor PE congruency.

The clinical and radiographic results at this series provided a good evidence for long-term efficacy of Corail stem with full extensive HA coating, which prevented most adverse events. HA-coating of stem appears to offer a satisfactory solution to fixation. However, polyethylene wear maybe a concern, especially in young patients.
